# Tandem gait performance in essential tremor patients correlates with cognitive function

**DOI:** 10.1186/s40673-014-0019-2

**Published:** 2015-01-10

**Authors:** Elan D Louis, Ashwini K Rao

**Affiliations:** GH Sergievsky Center, College of Physicians and Surgeons, Columbia University, New York, NY USA; Department of Neurology, College of Physicians and Surgeons, Columbia University, New York, NY USA; Department of Epidemiology, Mailman School of Public Health, Columbia University, New York, NY USA; Taub Institute for Research on Alzheimer’s Disease and the Aging Brain, College of Physicians and Surgeons, Columbia University, New York, NY USA; Program in Physical Therapy, Department of Rehabilitation & Regenerative Medicine, College of Physicians and Surgeons, Columbia University, New York, NY USA

**Keywords:** Essential tremor, Gait, Ataxia, Cognition, Cerebellum

## Abstract

**Background:**

Emerging yet separate literatures have highlighted gait/balance impairments (i.e., mild ataxia) and cognitive problems in patients with essential tremor (ET). However, the relationship between the two has not been studied. The goal of these analyses was to study the relationship between gait/balance impairments and cognitive problems in ET. One-hundred-twenty ET cases were enrolled in an epidemiological study at Columbia University Medical Center. A Telephone Interview for Cognitive Status (TICS, range = 0–41 [no deficits]) was administered and a videotaped assessment of tandem gait was performed, during which the number of missteps during 10-steps was counted.

**Results:**

The mean TICS score was 35.7 (range 25–39), and mean number of tandem mis-steps was 2.9 (range 0–10). The number of tandem mis-steps was correlated with the TICS score (Spearman’s r = −0.245, p = 0.011, i.e., individuals who had more tandem gait difficulty also had more cognitive difficulty). In a multivariate analysis, tandem mis-steps were associated with TICS score (p = 0.04) independent of age and other factors.

**Conclusions:**

More cognitive difficulty was associated with more tandem gait difficulty in ET. Ambulation often requires the concurrent use of both cognitive and motor neural systems; hence it is possible that the cognitive and gait problems in ET reflect an underlying pervasive disorder affecting both cognitive and motor circuits.

## Background

Essential tremor (ET) is a progressive neurological disease that has increasingly been linked with a dysfunction of the cerebellar system [1-5]. Aside from a variety of tremors, patients with (ET) may experience problems with gait and balance, exhibiting a mild form of gait ataxia [1]. Problems in tandem gait were the first type of gait problem to be noted in ET patients and since that time, tandem gait difficulty has been a consistent finding across many studies [6-8]. In some ET patients, the gait difficulty is marked [9]; indeed, the severity spectrum of the gait and balance disorder of ET has not been fully defined. Furthermore, its clinical correlates are not fully established. Functionally, the gait difficulty in ET has been linked with a loss of confidence in balance and it may result in near-falls and falls, particularly in the subgroup of ET patients with midline (i.e., cranial) tremors [8,10].

A range of cognitive problems have also been observed in ET cases, more so than in age-matched controls [11]. These include mild cognitive deficits (especially deficits in attention, executive function and several types of memory including working memory) as well as increased risks of mild cognitive impairment (MCI) and dementia [11-14]. The full impact of these problems is not fully understood.

Given the above, there is an increasing appreciation of ET as a complex cognitive-motor disorder [11]. Ambulation itself often requires the concurrent use of both cognitive and motor neural systems [15]. There is a growing literature examining the interaction between cognitive and motor function in the elderly and in those with a variety of neurological disorders. For example, cognitive impairment is an independent predictor of falls, increasing fall incidence for those with cognitive impairment to 80% [16]. Among individuals with MCI or Alzheimer’s Disease, a higher risk for falls is seen compared with healthy controls [17]. Yet the interplay between cognitive and motor function has received relatively little attention in ET [15]. Whether there is an association between cognitive and gait problems in ET has not been formally assessed. The goal of these analyses was to begin to study this association. The clinical relevance of these analyses is that in practice settings, it is important to identify those factors, and eventually those subgroups of patients, who are at greatest risk for difficulties with gait and balance and, by extension, risk for falls.

## Results and discussion

### Results: subject characteristics

There were 120 ET cases whose mean age was 71.3 ± 12.6 years (Table 1). The number of tandem mis-steps ranged from 0 – 10, with the mean being 2.9 ± 3.3 (Table 1). The mean Telephone Interview for Cognitive Status (TICS) score was 35.7 ± 2.3 (range = 25 – 39, Table 1).

**Table 1:** **Demographic and clinical characteristics of 120 ET cases**

Age (years)	71.3 ± 12.6
Female Gender	64 (53.3)
Education (years)	16.0 ± 3.0
Total tremor score	20.3 ± 5.6
Tremor duration (years)	30.9 ± 18.6
Age of tremor onset (years)	40.6 ± 20.2
Cranial tremor score^1^	
0	60 (50.8)
1	34 (28.8)
2	17 (14.4)
3	7 (5.9)
Tandem mis-steps	
0	33 (27.5)
1	24 (20.0)
2	16 (13.3)
3	15 (12.5)
4	6 (5.0)
5	6 (5.0)
6	0 (0.0)
7	2 (1.7)
8	1 (0.8)
9	1 (0.8)
10	16 (13.3)
Tandem mis-steps	2.9 ± 3.3 (range = 0 – 10)
TICS score	35.7 ± 2.3 (range = 25 – 39)

Values are mean ± standard deviation or number (percentage).

^1^Data were missing in two study subjects.

### Results: correlation of tandem gait with demographic and clinical variables

The number of tandem missteps was correlated with age (Spearman’s r = 0.46, p < 0.001, i.e., older age was associated with more gait difficulty) but not with years of education (Spearman’s r = −0.12, p = 0.19) or gender (Mann–Whitney test z = 0.94, p = 0.35). The number of tandem mis-steps was correlated with the cranial tremor score (Spearman’s r = 0.239, p = 0.009, i.e., individuals who had more gait difficulty also had more cranial tremor) but not with the total tremor score (Spearman’s r = 0.14, p = 0.15). The number of tandem mis-steps was not associated with the use of anticonvulsant medications (including primidone and phenobarbital), anxiolytic medications, or medications with sedative properties (Mann–Whitney tests, all three p > 0.32).

### Results: correlation of tandem gait with TICS score

The number of tandem mis-steps was correlated with the TICS score (Spearman’s r = −0.245, p = 0.011, i.e., individuals who had more gait difficulty also had more cognitive difficulty). We stratified the number of tandem mis-steps into quartiles (0 mis-steps [Quartile 1], 1 mis-step [Quartile 2], 2–3 mis-steps [Quartile 3], ≥4 mis-steps [Quartile 4]), and found that the TICS score declined with each increasing tandem mis-step quartile (mean TICS score = 36.4 ± 1.8, median = 37 [Quartile 1], mean TICS score = 36.2 ± 1.6, median = 36 [Quartile 2], mean TICS score = 35.7 ± 2.7, median = 36 [Quartile 3], and mean TICS score = 34.7 ± 2.7, median = 34.5 [Quartile 4], Jonckheere-Terpstra test p value = 0.009).

### Results: regression analyses

In a series of six bivariate linear regression analyses, log-transformed tandem mis-steps were associated with TICS score (beta = −0.043, p = 0.001), age (beta = 0.013, p < 0.001), and cranial tremor score (beta = 0.11, p = 0.002), but not with use of anticonvulsant medications (including primidone and phenobarbital), anxiolytic medications, or medications with sedative properties (all three p > 0.25).

In a multivariate linear regression analysis in which all six variables were entered, log-transformed tandem mis-steps were *independently* associated with TICS score (beta = −0.03, p = 0.04, i.e., more cognitive difficulty was associated with more gait difficulty), age (beta = 0.10, p < 0.001, i.e., older age was associated with more gait difficulty), and cranial tremor score (beta = 0.069, p = 0.05, i.e., more extensive cranial tremor was associated with greater gait difficultly) but none of the other variables (all p values > 0.05).

### Discussion

A number of clinical aspects of ET are currently under increased scrutiny. These include various non-motor features as well as motor features other than tremor. Hence, there is an increasing appreciation of ET as a complex cognitive-motor disorder [11]. The nature of the cognitive impairment in ET is not entirely clear, but could involve processes restricted to the cerebellum as well as those outside of the cerebellum [18,19]. The interplay between cognitive and motor function has received relatively little attention in ET [11]. The current data demonstrate an interaction between these functions in ET patients, indicating that they do not affect patients in isolation.

Our results are comparable with literature examining the interaction between cognitive and motor function in the elderly and in those with other neurological disorders. Thus, cognitive impairment is an independent predictor of falls, increasing fall incidence for those with cognitive impairment to 80% [16]. In individuals with primary impairment of cognition, such as MCI or Alzheimer’s Disease, a higher risk for falls is seen compared with healthy controls [17]. Impairments in cognition may limit attention resources that need to be allocated for safe functional ambulation.

The nature of the interaction is not clear. Ambulation often requires the concurrent use of both cognitive and motor neural systems [15]; hence it is possible that the cognitive and gait problems in ET reflect an underlying pervasive disorder affecting both cognitive and motor circuits. Alternatively, the effects of cognitive dysfunction in ET might extend to the performance of functional-motor activities, pointing towards another dimension of the cognitive issues that occur in patients with ET. Further study is needed.

We observed a median 2.5 point difference in TICS scores between the highest and lowest tandem mis-steps quartiles; hence the difference was clinically modest. Indeed, in our adjusted analyses, age was a stronger predictor of tandem mis-steps than TICS score. This association with age was not a surprising finding as many aspect of motor function, including gait, decline with advancing age among elderly patients.

As in our earlier study, conducted on data collected during the baseline rather than follow-up phase of this study, patients with more extensive cranial tremor also demonstrated greater difficulty with tandem gait, suggesting that midline cerebellar structures might be involved with both the cranial tremor and gait impairments [8]. The correlates of gait difficulty in ET are likely to be multi-fold and complex.

This study had limitations. First, a more extensive cognitive test battery would have helped to further define and refine our understanding of the links between cognitive function, and specific cognitive domains, and functional gait difficulty in ET. However, a detailed cognitive test battery was not a feature of this study. Even with this limitation, we were still able to detect a significant correlation in this study. Second, a more extensive gait battery would similarly have helped to further define these links. This study also had several strengths, including the uniform study assessment across patients, and large sample size as well as careful adjustment for medication effects.

## Conclusions

In summary, performance on a cognitive screen in ET was associated with performance on a functional gait task, such that more cognitive difficulty was associated with more gait difficulty. These results add another dimension to the cognitive dysfunction of ET, indicating that the effects of cognitive impairments in ET might extend to the performance of functional-motor activities. In clinical practice settings, this suggests that patients with ET who are experiencing cognitive deficits might be particularly prone to difficulties with gait and balance.

## Methods

### Subjects

ET cases were enrolled in an ongoing clinical-epidemiological longitudinal study of ET at the Neurological Institute, Columbia University Medical Center (CUMC), as described elsewhere [8,20]. The study assessed a wide range of clinical features as well as the role of environmental factors in disease etiology. The participants were adults (age 18 and older) derived from two primary sources: (1) patients whose neurologist was on staff at the Institute or (2) patients who were cared for by their local doctor in the tri-state region (New York, New Jersey, Connecticut) and, as members of the International Essential Tremor Foundation, had read advertisements for the study and volunteered. Prior to enrollment, all cases signed informed consent approved by the CUMC Ethics Committee. After enrollment, all diagnoses were confirmed using published diagnostic criteria, as outlined below. Baseline recruitment began in 2000 and ended in 2009, during which more than 300 ET cases were enrolled. In April 2009, a follow-up phase began, with the goal of enrolling at least 120–130 ET cases. During the follow-up phase, the oldest cases were targeted first (i.e. those with the highest likelihood of loss to follow-up due to mortality). During recruitment for the follow-up phase, 41 ET cases (24 [58.5%] women, 17 men; age 75.4 ± 13.0 years) refused to participate. At follow-up, ET enrollees underwent the same in-person evaluation as at baseline, with a few modifications, including an assessment of tandem gait (see below).

### Evaluation

The data were derived from the first-follow-up visit (2009 – 2013), during which an assessment of tandem gait (see below) was uniformly incorporated into the study protocol.

During this assessment, a trained research assistant administered a demographic and medical history, including years of education beginning with first grade. Data on all current medications were collected. As the study required the valid completion of questionnaires, a Telephone Interview for Cognitive Status (TICS, range = 0 – 41 [no deficits]) was administered [21] as a brief cognitive screen on the same day as the remainder of the assessment; it was administered in person. This tool was used because it was the same tool that had been used as baseline, although then it had been administered by telephone. TICS has very good sensitivity and specificity for the diagnosis of Alzheimer’s disease and high test-retest reliability. It has been shown to have sufficient range to be useful in field research studies of Alzheimer’s disease and other disorders associated with cognitive impairment. It is composed of 11 items (maximum score = 41), and assesses orientation, attention, memory, working memory/calculation, and language (i.e., several of the domains noted to be affected in patients with ET). A score ≥ 31 distinguishes between normal and mild dementia (94% sensitivity, 100% specificity) [21].

An assessment of tandem gait was performed in all study subjects who participated during the study visit and it was videotaped so that the number of mis-steps could be evaluated later by a senior neurologist (E.D.L.). Tandem gait was explained and demonstrated to subjects; they were carefully instructed to walk placing one directly foot in front of the other, being careful to touch toe to heel with each step. If they mis-understood the task (i.e., failed to follow directions), they were immediately re-instructed and began again. They could chose their own line (i.e., a line was not drawn or placed on the floor). The number of missteps (i.e., steps to the side) during a single trial of 10-steps was counted (E.D.L.).

During the assessment, a videotaped neurological examination was also performed. This included one test for postural tremor and five for kinetic tremor (pouring, using spoon, drinking, finger-nose-finger, drawing spirals) performed with each arm (12 tests total). A neurologist specializing in movement disorders (E.D.L.) used a reliable [22] and valid [23] clinical rating scale, the Washington Heights-Inwood Genetic Study of ET (WHIGET) tremor rating scale, to rate postural and kinetic tremor during each test: 0 (none), 1 (mild), 2 (moderate), 3 (severe). These ratings resulted in a total tremor score (range = 0 – 36), which is an assessment of postural and kinetic tremor [24]. Diagnoses of ET were re-confirmed by E.D.L. using the videotaped neurological examination as well as the WHIGET diagnostic criteria (moderate or greater amplitude kinetic tremor [tremor rating ≥ 2] during three or more tests or a head tremor, in the absence of Parkinson’s disease, dystonia or another cause) [24]. The WHIGET diagnostic criteria for ET were developed for a population-based genetic study and, based on data from approximately 2,000 normal (non-diseased controls), these criteria carefully specify the specific examination maneuvers during which tremor should be present and the severity of tremor that should be evident during these maneuvers. These criteria have been shown to be both reliable [22] and valid [24]. The WHIGET criteria have been used routinely in Dr. Louis’ epidemiological studies of ET [25-29] and are used by other tremor investigators in the US and internationally [30-39].

On videotaped examination, jaw and voice tremors were coded as present or absent while cases were seated facing the camera. Jaw tremor was assessed while the mouth was stationary (closed), while the mouth was slightly open, during sustained phonation, and during speech. Voice tremor was assessed during sustained phonation, while reading a prepared paragraph, and during speech. Neck tremor in ET was coded as present or absent and was distinguished from dystonic tremor by the absence of twisting or tilting movements of the neck, jerk-like or sustained neck deviation, or hypertrophy of neck muscles; it was distinguished from titubation by its faster speed and the absence of accompanying truncal titubation or ataxia while seated or standing. As described elsewhere, a cranial tremor score (range = 0 – 3) was calculated for each subject based on the number of locations (jaw, voice, neck) in which tremor was present on examination [8].

### Statistical analyses

The number of tandem mis-steps was not normally distributed (Kolmogorov-Smirnov test p value < 0.001). Therefore, the correlation between number of tandem mis-steps and TICS score as well as other variables (age, gender, education, cranial tremor score, total tremor score, use of anticonvulsant medications, use of anxiolytic medications, use of medications with sedative properties) was assessed using non-parametric tests (Spearman’s correlation coefficients and Mann–Whitney tests). In one analysis, we stratified the number of tandem mis-steps into quartiles; for that analysis we stratified the TICS score into tertiles because the range of TICS scores was not sufficient for quartiles. We then examined the association across these categories using a Jonckheere-Terpstra test. As previously described [8], for linear regression models, we modified the tandem mis-step variable - because in many cases, the number of tandem mis-steps was zero and the number of mis-steps was not normally distributed, we used the following value: log_10_ (tandem mis-steps +1). First, we examined a series of six bivariate models, assessing variables that were associated with tandem mis-steps in our initial analyses. We also assessed medications, as these have a high probability of being associated with tandem mis-steps, regardless of initial findings in the dataset. Then, a multivariate model, all of the variables considered into the bivariate models were entered at the same time.

## Ethics approval and consent to participate

Prior to enrollment, all cases signed informed consent approved by the CUMC Ethics Committee.

## Abbreviations

CUMC: Columbia University Medical Center
ET: Essential tremor
MCI: Mild cognitive impairment
TICS: Telephone Interview for Cognitive Status
WHIGET: Washington Heights-Inwood Genetic Study of ET
